# HRGPred: Prediction of herbicide resistant genes with *k*-mer nucleotide compositional features and support vector machine

**DOI:** 10.1038/s41598-018-37309-9

**Published:** 2019-01-28

**Authors:** Prabina Kumar Meher, Tanmaya Kumar Sahu, K. Raghunandan, Shachi Gahoi, Nalini Kanta Choudhury, Atmakuri Ramakrishna Rao

**Affiliations:** 1 Division of Statistical Genetics, ICAR-Indian Agricultural Statistics Research Institute, New Delhi, 110012 India; 2 Centre for Agricultural Bioinformatics, ICAR-Indian Agricultural Statistics Research Institute, New Delhi, 110012 India; 3 Division of Genetics, ICAR-Indian Agricultural Research Institute, New Delhi, 110012 India

## Abstract

Herbicide resistance (HR) is a major concern for the agricultural producers as well as environmentalists. Resistance to commonly used herbicides are conferred due to mutation(s) in the genes encoding herbicide target sites/proteins (GETS). Identification of these genes through wet-lab experiments is time consuming and expensive. Thus, a supervised learning-based computational model has been proposed in this study, which is first of its kind for the prediction of seven classes of GETS. The cDNA sequences of the genes were initially transformed into numeric features based on the *k*-mer compositions and then supplied as input to the support vector machine. In the proposed SVM-based model, the prediction occurs in two stages, where a binary classifier in the first stage discriminates the genes involved in conferring the resistance to herbicides from other genes, followed by a multi-class classifier in the second stage that categorizes the predicted herbicide resistant genes in the first stage into any one of the seven resistant classes. Overall classification accuracies were observed to be ~89% and >97% for binary and multi-class classifications respectively. The proposed model confirmed higher accuracy than the homology-based algorithms viz., BLAST and Hidden Markov Model. Besides, the developed computational model achieved ~87% accuracy, while tested with an independent dataset. An online prediction server HRGPred (http://cabgrid.res.in:8080/hrgpred) has also been established to facilitate the prediction of GETS by the scientific community.

## Introduction

The genetic ability of a weed biotype to survive after being treated with a lethal dose of herbicide and reproduce normally is defined as herbicide resistance (HR) (http://www.hracglobal.com/). The HR is a serious threat to the sustainable food production, worldwide^1^. In fact, HR has incurred higher crop yield loss than any other crop pest species^2^. The HR in crops evolved as a result of intense selection pressure exerted by the frequent and wide-spread application of herbicides^3,4^. The target-site-resistance-mechanism (TSRM) and non-target-site-resistance-mechanism (NTSRM) are the two major categories of HR mechanisms^5^. The TSRM is mainly associated with the mutation(s) in the genes encoding herbicide target sites/proteins (GETS) that results non-binding of herbicides to the site of action and thus prevents the phytotoxicity of the herbicides^6^. In contrast to TSRM, NTSRM is normally related to the biochemical modification of herbicides (detoxification) and/or sequestration of the herbicides and their metabolites to other parts of the plant cells^7,8^. In resistant weed species, the TSRMs were widely reported as compared to the NTSRMs^9^. In particular, HR mechanisms for seven classes of GETS viz., ALS (acetolactate synthase), GS (Glutamine synthetase), EPSPS (5-enolpyruvylshikimate-3-phosphate synthase), PPO (Protoporphyrinogen oxidase), ACCase (Acetyl-CoA carboxylase), HPPD (4-Hydroxyphenylpyruvate dioxygenase) and PDS (Phytoene desaturase) have been reported in literatures.

Resistance conferred to various herbicides due to mutation(s) in the GETS has been well studied for the above seven categories of target enzymes. Specifically, several weed biotypes were reported to confer resistance to the ALS inhibiting herbicides, when substitution takes place in any of the amino acids Ala205, Ala122, Asp376, Pro197, Ser653 and Trp574n^10^. The gene PPX2L with a deleted codon in the biotype of *A. tuberculatus* has been confirmed to be involved with the PPO inhibiting HR^11^. The mutations Ile-2041-Asn and Trp-574-Leu in *L. multiflorum* conferred resistance to inhibitors of ALS and ACCase respectively^12^. Further, mutation in GS conferring resistance to herbicide glyfosinate was identified by Pornprom *et al*.^13^. A point mutation, P106L in EPSPS conferred resistance to glyphosate in *Eleuisine indica*^14^ and *Lolium rigidum*^15^. High level of resistance was conferred to glycophosphte in *E. indica* because of two amino acid substitutions (T102I + P106S) in EPSPS^16^. Three somatic mutations at codon position 304 of PDS enzyme have been reported to confer resistance against herbicide fluridon in *hydrilla verticillata*^17,18^. Resistance to norflurazon herbicide in *Chlamydomonas reinharditi* was also due to a mutation (F131V) in the PDS enzyme^19^. Further, the point mutation in PDS also made *Chlorella zofingiensis*^20^ and *H. pluvialis*^21^ norflurazon resistant. Though the resistance to mesotrione in *A. palmeris* was primarily due to the herbicide detoxification^22^, higher expression of HPPD gene contributing to the resistance has also been reported by Nakka *et al*.^23^.

Understanding the evolution of HR is becoming simpler with the advancement in molecular biology^24^. Transcriptome profiling analysis has help enabled the identification of HR associated genes. As evidenced from literatures, genes mainly involved in NTSRM have been identified by transcriptome profiling than the genes involved in TSRM. For instance, transcriptome profiling studies have been performed to identify the genes as far as the resistance to diclofop in *L. Rigidium*^25^ and paraquat in goose grass^26^ is concerned. Similar analyses have also been carried out to understand the glyphosate resistance mechanism in giant ragweed^27^, and mesosulfuron-methyl & fenoxaprop-P-ethyl resistance mechanism in a short awn foxtail population^28^. Recently, Babineau *et al*.^29^ have identified transcripts and gene families associated with the metabolic-based HR in *A. spica-venti*, based on a *de novo* transcriptome analysis.

Genetic factors (mutations) have been known to be associated with the evolution of HR. But, it is very difficult to predict and identify the biotypes which will develop resistance to a specific chemical class^30^. Nevertheless, accurate prediction of the herbicidal activities and sites of action for new chemical classes without extensive laboratory experiments would be highly beneficial^31^. Moreover, identifying the genes conferring resistance to different chemical classes in wet-lab is resource intensive. Thus, an attempt has been made in this study to computationally identify the seven classes of genes involved in the TSRM. We believe that the developed computational model will be helpful for reliable prediction of the seven classes of GETS.

## Material and Methods

Many computational studies^32–38^ in the recent past have adopted five guidelines for developing supervised learning model-based predictor. The guidelines are given below.

(i) Prepare datasets of highest standard for training and evaluating the predictor comprehensively.
(ii) Transform the sequence dataset (DNA/RNA/Protein) into numeric form by using such an encoding scheme which can reflect maximum correlation with the concerned target.
(iii) Propose a competent prediction algorithm.
(iv) Employ proper validation approach to measure the efficiency of the developed computational model.
(v) Built a freely accessible prediction server using the developed approach for the benefit of scientific community.

We have also followed the above mentioned guidelines, where the steps are described one-by-one in the following sections.

### Acquisition of herbicide resistant and non-resistant sequence datasets

First of all, 227 cDNA sequences for all the seven categories of GETS (36 EPSPS, 31 GS, 45 AACase, 46 ALS, 22 HPPD, 25 PPO and 22 PDS) were collected from the herbicide resistant weeds database (http://www.weedscience.org/Sequence/sequence.aspx). These 227 sequences of the resistant category were found to be distributed over 87 herbs. Out of 227, 20% sequences from each resistant category (7 EPSPS, 6 GS, 9 AACase, 9 ALS, 4 HPPD, 5 PPO and 4 PDS) was taken to construct the independent test set for the resistant class and the remaining 183 sequences were included in the positive set (resistant class) for model evaluation. Further, sequences with >90% pair-wise sequence identities were also removed from the positive set by using CD-HIT^39^ program to avoid homologous bias. A total of 122 resistant sequences (obtained after removing redundancy) were considered to build the final positive dataset for model evaluation. For preparing the negative dataset (non-resistant class), the following steps were followed.

(i) The cDNA sequences from the same 87 herbs (excluding the sequences present in the resistant class) were collected from the NCBI. For the species *Alnus glutinosa, Nicotiana benthamiana*, *Raphanus raphanistrum, Glycine max*, *Zea mays* and *Triticum aestivum* a large number of sequences were obtained and therefore excluded to avoid the computational complexity and 3292 sequences belonging to the remaining species were retained.
(ii) Then, the sequences having non-standard bases as well as annotated with partial CDS were also removed and 2282 sequences were obtained (out of 3292).
(iii) Further, the sequences with >60% pair-wise sequence identities were removed from 2282 sequences using CD-HIT program, to avoid homologous bias. Finally, 1444 sequences obtained after redundancy check were used to make the negative dataset (non-resistant class).

So, the final dataset containing 122 resistant and 1444 non-resistant sequences was used for evaluation of the model through cross validation procedure.

### Feature generation

Mapping of input biological sequences onto numeric feature vectors is the first and foremost requirement before using them as an input in the supervised learning algorithms. Since oligomer frequencies have been widely and successfully used as features to model the functions and properties of biological sequences (DNA, RNA and protein), these frequencies were also used in this work. Here, two different types of *k*-mer feature viz., contiguous *k*-mer (CkM) and pseudo *k*-mer (PkM) were employed. The CkM features have been used earlier for classifying the bacterial genome^40^, biological sequence clustering^41^, predicting splicing junctions^42^, DNA barcode-based species identification^43^ and in other studies. Also, the PkM features have been successfully used in many bioinformatics studies such as identification of DNA methylation sites^44^, protein-protein interaction^45^, N6-methyladenosine sites^46^, RNA 5-methyl cytosine sites^47^ and prediction of protein sub mitochondrial locations^48^. Computational procedures of these features are precisely described below.

Let *D* (*X*_1_*X*_2_*X*_3_…*X*_*N*_) be any DNA sequence with *N* nucleotides, where *X*_*l*_ denotes the nucleotide (A/T/G/C) at *l*^th^ position in the sequence. Then, based on the CkM features each sequence can be represented with a numeric vector of 4^k^ components ${f}_{1},\,{f}_{2},\,{f}_{3},\,\mathrm{...},\,{f}_{{4}^{k}}$, where *f*_*i*_ represents the frequency (normalized) of the *i*^th^ *k*-mer. For the large size of *k*-mer, the effects of the sequence order within a short range can be easily accounted but it is difficult to account the global sequence-order information. Therefore, with an aim to improve the accuracy by incorporating the information of global sequence-ordering, Guo *et al*.^49^ proposed a new sequence encoding scheme known as PkM compositions, which is similar to the pseudo-compositions of nucleotides proposed by Chou^50^. The PkM features of a DNA sequence can be encapsulated with a (4^*k*^ + *d*)-dimension numeric vector ${{v}}_{1},{{v}}_{2},\,\ldots \,{{v}}_{{4}^{k}},\,{{v}}_{{4}^{k}+1},\,\ldots ,\,{{v}}_{{4}^{k}+{\rm{d}}}$, where *v*_*x*_ is given by

$$\{\begin{array}{c}\frac{{f}_{x}}{\sum _{i=1}^{{4}^{k}}{f}_{i}+w\sum _{j=1}^{{4}^{k}}{\rho }_{j}},\,\,1\le x\le {4}^{k}\\ \frac{w{\rho }_{x}-{4}^{k}}{\sum _{i=1}^{{4}^{k}}{f}_{i}+w\sum _{j=1}^{{4}^{k}}{\rho }_{j}},\,\,{4}^{k}+1\le x\le {4}^{k}+d\end{array}.$$

Here, *d*, *f*_*i*_ and *w* respectively represent the tier of correlation, normalized frequency of the *i*^th^ *k*-mer and weight factor. Further, *ρ*_*j*_ denotes the *j*^th^-tier correlation between all the *j*^th^ most CkM, where ${\rho }_{j}=\frac{1}{N-j-k+1}\sum _{i=1}^{N-j-k+1}{{\rm{\Omega }}}_{i,i+j}\,;\,j=1,2,\,\mathrm{...},\,d( < N-k)$. The Ω_*i*,*i* + *j*_ is the correlation function represented by ${[p({X}_{i}{X}_{i+1}\ldots {X}_{i+k-1})-p({X}_{i+j}{X}_{i+j+1}\ldots {X}_{i+j+k-1})]}^{2},$ where $p({X}_{i}{X}_{i+1}\mathrm{...}{X}_{i+k-1})$ and $p({X}_{i+j}{X}_{i+j+1}\mathrm{...}{X}_{i+j+k-1})$ are the proportions of *k*-mers *X*_*i*_*X*_*i*+1_…*X*_*i*+*k*−1_ at pos*i*tions *i* and *X*_*i*+*j*_*X*_*i*+*j*+1_…*X*_*i*+*j*+*k*−1_ at pos*i*tions *i* + *j* respectively. In this work, we have considered 1^st^ tier correlation only. Moreover, CkM and PkM features were computed for the *k*-mer sizes 1, 2, 3 and 4. Thus, a total of 340 and 344 descriptors were generated for CkM and PkM feature sets respectively.

### Support vector machine

Because of sound statistical background and non-parametric nature, the support vector machine (SVM)^51^ has been successfully employed in numerous biological studies including bioinformatics^52,53^ and computational biology^54–57^. Ability to handle the large and noisy input dataset is also one of the reasons for wide and successful application of SVM in several computational studies^58,59^. Performance of SVM varies with the use of different kernel functions that transform the input dataset to the feature space of high dimension, where the optimal separating hyper-plane linearly separates the observations belonging to different classes. For the given input vectors **x** s with class level *y* ∈ {*resistant*, *non* − *resistant*}, the decision function in SVM is $f({\bf{x}})=sign[\sum _{i=1}^{N}{\alpha }_{i}{y}_{i}K({\bf{x}},\,{{\bf{x}}}_{i})+b]$. The coefficients *α*_*i*_ s are obtained by solving the convex quadratic programming $\mathop{{\rm{\max }}}\limits_{\alpha }(-\frac{1}{2}\sum _{i,j=1}^{N}{\alpha }_{i}{\alpha }_{j}{y}_{i}{y}_{j}K({{\bf{x}}}_{i},\,{{\bf{x}}}_{j})+\sum _{i=1}^{N}{\alpha }_{i})$ subject to the conditions 0 ≤ *α* _*i*_≤ *C* and $\sum _{i=1}^{N}{\alpha }_{i}{y}_{i}=0$, where *C* is the regularization parameter. Higher value of *C* generates smaller margin that results in few misclassification of training examples. On the other hand, smaller value of *C* produces margin of larger width that leads to more misclassification error of the training set. In other words, samples inside the margin contributing to the overall misclassification error are less penalized with the lower value of *C* than the higher one. With the value of *C* as 0, samples inside the margins are not penalized, and the default value of *C* is 1. We have used four commonly used kernels (K) in this work that are as follows:

$$K({{\bf{x}}}_{i},\,{{\bf{x}}}_{j})=\{\begin{array}{c}{{\bf{x}}}_{i}^{T}{{\bf{x}}}_{j}\,(Linear\,kernel)\\ {(\gamma {{\bf{x}}}_{i}^{T}{{\bf{x}}}_{j}+r)}^{d}\,(Polynomial\,kernel\,of\,degree\,d)\\ \exp \{-\gamma {\Vert {{\bf{x}}}_{i}-{{\bf{x}}}_{j}\Vert }^{2}\}\,(RBF\,kernel)\\ \tanh \,(\gamma {{\bf{x}}}_{i}^{T}{{\bf{x}}}_{j}+r)\,(Sigmoid\,kernel\,)\,\end{array}$$

Here *r*(*bias*), *d*(*degree*) and *γ*(*gamma*) are the kernel parameters with default values 0, 3 and 1/(dimension of data) respectively. For the larger value of gamma (kernel width), radius of the area of influence of the support vectors only includes the support vector itself and any changes in the value of *C* will not be able to prevent from over-fitting of the model. The bias term *r* compensates the feature vectors that are not centred around zero. In other words, if the features are centred around zero the bias term isn’t always needed. The flexibility of the decision boundary in case of the polynomial kernel depends upon degree (*d*), where a higher degree produces a more flexible decision boundary. In this work, using a sample dataset of 50 resistant and 50 non-resistant observations, the kernel with the highest accuracy was first identified among all the four kernels using default parametric values. The R-package *e1071*^60^ was utilized to implement the SVM model. Also, the same sample dataset was utilized to choose the feature set with the highest accuracy between CkM and PkM feature sets.

### Validation of the developed approach

We adopted K-fold cross-validation (CV), jackknife validation and independent data set validation for assessing the performance of the established predictor^61^. Five-fold CV was employed for evaluating the binary classifier, whereas the jackknife validation was adopted for assessing the multi-class classifier. In addition, the developed computational model was also evaluated with a blind (independent) dataset that was neither utilized as training nor as test set. For performing 5-fold CV, five equal-sized subsets were prepared having same number of observations randomly drawn from both the classes in each subset. In each fold, four subsets were utilized for training of the model and the rest one subset was utilized as the test set for validating the accuracy of the respective trained model. Here, each subset was tested exactly once while the procedure was repeated five times. In case of jackknife validation, one observation was singled out every time and predicted by the model that was built with the remaining observations.

### Performance measure

The performance metrics viz., Sen (Sensitivity), Spe (Specificity), Acc (Accuracy), Pre (Precision), MCC (Matthew’s correlation coefficient), AUC-ROC (area under ROC curve)^62^ and AUC-PR (area under precision-recall curve)^63^ were considered for measuring performance of the newly established computational model. The metrics are defined as follows:

$$\begin{array}{rcl}Sen & = & tp/(tp+fn);\,Spe=tn/(tn+fp);\,Acc=(tp+tn)/(tp+fn+tn+fp)\\ MCC & = & ((tp\times tn)-(fp\times fn))/\sqrt{(tp+fp)\times (tp+fn)\times (tn+fp)\times (tn+fn)}\\ Pre & = & tp/(tp+fp).\end{array}$$

In the above metrics, *tp (true positive)*, *tn (true negative)*, *fn (false negative)* and *fp (false positive)* respectively denotes the number of observed resistant sequences, non-resistant sequences, resistant sequences misclassified as non-resistant and non-resistant sequences misclassified as resistant. The MCC measures the correlation between the predicted and real results^64^. Its value lies between −1 and +1, where +1 represents an accurate prediction and −1 demonstrates a complete disagreement between the predicted and observed results. The MCC of 0 means a random prediction^65^. The ROC curves can be plotted by taking false positive (*fp*) and true positive (*tp*) rates in x- and y- axes respectively, with varying thresholds. The range of AUC-ROC lies between 0 and 1, where an AUC-ROC of 1, 0.5 and <0.5 imply an accurate, a random and an under-performed classifier respectively^64,65^. Unlike ROC, PR curve takes into account the class distribution. The PR curve can be obtained by plotting the precision and recall in x- and y- axes respectively, where the range of AUC-PR is 0 to 1 with a value close to 1 represents a better classifier.

### Binary classification using balanced datasets

Size of the non-resistant class is overwhelmingly larger than that of resistant class, and hence the dataset is highly unbalanced. Machine learning-based classifier may produce biased result towards the major (non-resistant) class, if such unbalanced dataset is used^44^. Therefore, the binary classification was performed using the balanced dataset having approximately same number of observations from each class. In particular, the balanced dataset was prepared by randomly drawing 120 sequences from both the resistant and non-resistant classes. Moreover, the developed computational method was evaluated over 100 such bootstrap sets, where each set contains 120 resistant and 120-non resistant sequences drawn at random from the respective classes. In each bootstrap set, performance was assessed through 5-fold CV, and the performance metrics were computed by taking the average over all the 100 bootstrap sets.

### One-to-one discrimination

Here, classification was made among seven categories of GETS. Before using the sequences for classification, homologous bias was removed in each resistant category by excluding those sequences which were >90% similar with any other sequences of that category. Finally, a dataset consisting of 36 ACCase, 37 ALS, 29 EPSPS, 25 GS, 18 HPPD, 18 PDS and 20 PPO was prepared. Since size of the dataset is small for each category, jackknife validation was employed to assess the performance of the computational model.

### Two-stage prediction for independent test set

Motivated by the earlier works^38,66–69^, prediction of the test instances was made in two stages. In the first stage, a binary classifier (trained with resistant and non-resistant classes) predicts the test sequences as either herbicide resistant or non-resistant. In the second stage, a multi-class classifier (trained with seven resistant classes) categorizes the sequences (that were predicted in the resistant class in the first stage) to any one of the seven resistant categories.

### Comparison with BLAST algorithm for binary classification

Performance of the developed computational method was also compared with that of homology-based method BLAST^70^. Three types of nucleotide blast viz., *blastn*, *megablast* and discontinuous megablast (*dc-megablast*) available at NCBI^71^ were used. Comparison was made using the performance metrics computed over five folds of CV. For performing CV, the blast software of NCBI was installed in a local server and executed in an offline mode. In each fold, database was built with the resistant class of the training set and the respective test set (consisting of sequences from both resistant and non-resistant classes) was designated as query. Depending upon the similar sequences obtained from the blast search, each test (query) instance was assigned to either resistant or non-resistant class. Here, the BLAST analysis was carried out using the e-values 0.1, 1. Besides, performance of the BLAST algorithms were also assessed using different word size (K-mer). In particular, *blastn* analysis was performed with word sizes 8, 9, 10, 11(default), 12, 13 and 14, *megablast* was carried out with word sizes 8, 9, 10, 11 (default), 12, 13 and 14, and *dc-megablast* was evaluated with the two possible word sizes 11 and 12.

### Comparison with Hidden Markov Model (HMM) for binary classification

It has been established that HMM often captures more information and produces reliable results than the BLAST. Thus, performance of the HMM was also assessed with the same dataset that was used for evaluating the BLAST algorithms. The HMM was executed using the standalone version of HMMER 3.1b2^72^. In each fold of the 5-fold CV procedure, HMM profile was created using the resistant class of the training set with the help of the module *hmmbuild*. The query dataset having sequences from both resistant and non-resistant classes was then searched against the created HMM profile using the *hmmsearch* module, with different parameter combinations i.e., e-value (0.1, 0.01 and 0.001), controlling priors (*pnone* and *plaplace*), effective sequence weight (*eent*, *eclust* and *enone*) and relative sequence weight (*wpb*, *wgsc* and *wblosum*). Detailed descriptions about these parameters can be seen from the HMMER site (http://hmmer.org/).

### Comparison with other supervised learning techniques for binary classification

Predictive ability of the developed computational model was also compared against other supervised learning models viz., artificial neural network (ANN)^73^, AdaBoost^74^, Bagging^75^ and Random Forest (RF)^76^. Comparisons among the models were made using the metrics computed over 5-folds of CV. The R-packages *randomForest*^77^ (function: *randomForest*), *RSNNS*^78^ (function: *mlp*), *ada*^79^ (function: *ada*) and *ipred*^80^ (function: *bagging*) were used for implementing the RF, ANN, AdaBoost and Bagging classifiers respectively.

### Development of prediction server

As stated by Chou and Shen^81^, development of a freely accessible user-friendly web server has always been a future direction for establishment of other computational tools. Moreover, development of such tool will significantly enhance the impact of any theoretical work. Keeping this in mind, we have established a web server based on the developed computational model for predicting GETS by the experimental scientists as well as other stake holders. The front end of the server was designed using Hypertext Mark-up Language (HTML) and Hypertext Pre-processor (PHP). Besides, a developed R-program was implemented at the back end through PHP. To run the server, user has to submit the cDNA sequence(s) in FASTA format.

## Results

### Feature and kernel analysis

**Figure 1:**
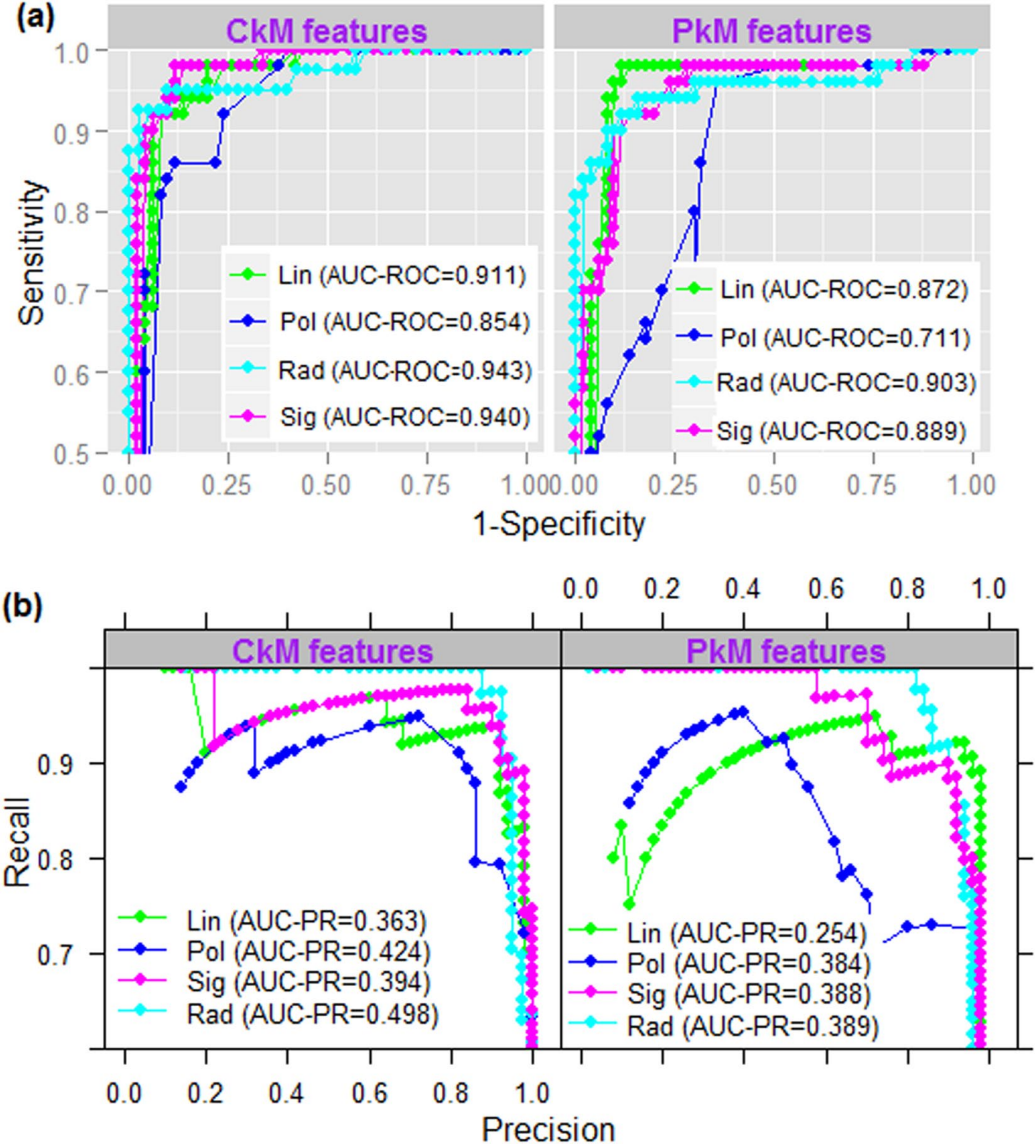
(**a**) ROC curves and (**b**) PR curves of SVM, under different feature-kernel combinations. It can be seen that the AUC-ROC and AUC-PR values are higher for the radial kernel under CkM feature set than that of other feature-kernel combinations.

Under RBF kernel (with default parameters), AUC-ROC for PkM and CkM features are observed to be 0.903 and 0.943 respectively (Fig. 1a), which are higher than that of other three kernels (Fig. 1a). Similarly, AUC-PR for PkM (0.389) and CkM (0.498) feature sets under RBF are also higher than the other kernels (Fig. 1b). Further, AUC-PR and AUC-ROC are higher for CkM as compared to the PkM feature set. Since higher accuracies are found for CkM feature set under RBF kernel, the same feature-kernel combination is preferred in the subsequent analysis. Using the same sample dataset, the regularization parameter *C* (2^−5^ to 2^15^ with step 2) and the kernel width parameter γ (2^−15^ to 2^−5^ with step 2^−1^) for RBF kernel were further optimized using the *tune. svm* function available in the same package. The optimum values of *C* and *γ* are seen almost equal with their default parametric values 1 and 0.0029 (1/340) respectively.

### Analysis of CkM features

**Figure 2:**
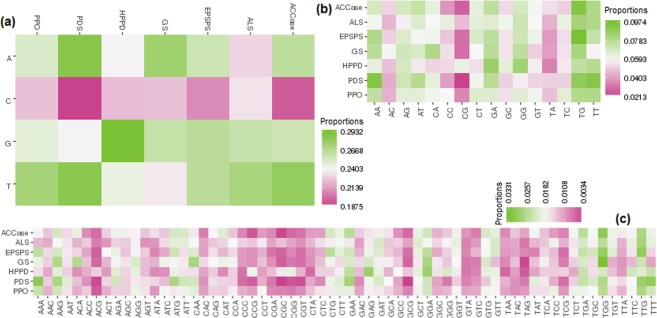
Heat maps for (**a**) nucleotides, (**b**) di-nucleotides and (**c**) tri-nucleotides compositions.

It is observed that the proportions of nucleotide C are less as compared to A, T and G in all the resistant categories (Fig. 2a). As far as di-nucleotides are concerned, AA, AG, AT, CA, CT, GA, GG, TG and TT occurred more frequently than that of AC, CC, CG, GC, GT, TA and TC (Fig. 2b). Similarly, occurrence probabilities are found to be higher for AAG, ATG, ATT, CAA, CTG, CTT, GAG, GAT, GCT, GGA, GGT, GTG, GTT, TGA, TGC, TGG, TTC, TTG and TTT as compared to the other tri-nucleotides (Fig. 2c).

**Figure 3:**
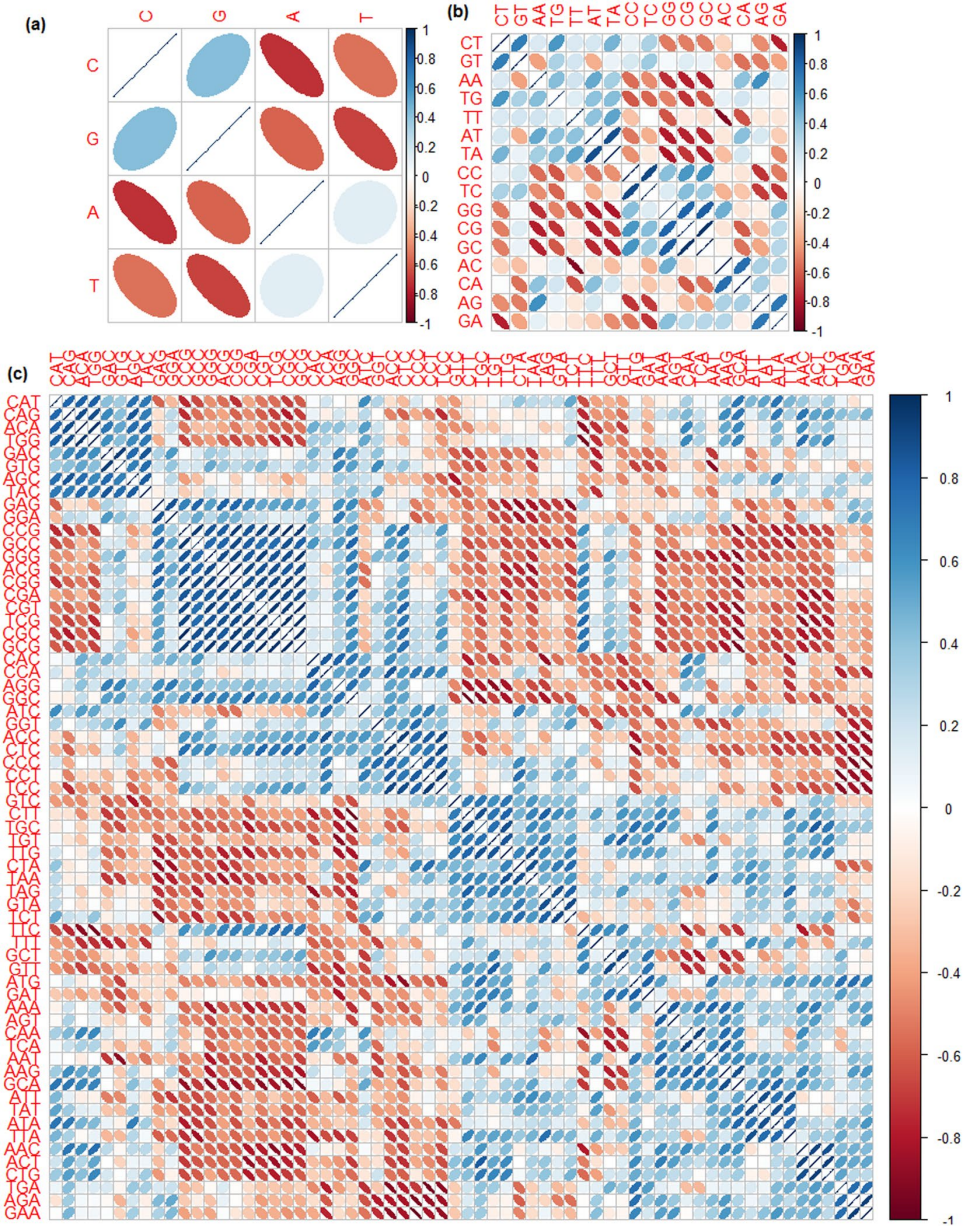
Heat maps of the correlations among (**a**) four nucleotides, (**b**) sixteen di-nucleotides and (**c**) sixty four tri-nucleotides.

From Fig. 3a, it is seen that the occurrences of A and T are negatively correlated with that of G and C, whereas the correlation is positive between the occurrences of A and T as well as between the occurrences of G and C. Further, positive correlations are found among the occurrences of di-nucleotides CT, GT, AA, TG, TT, AT and TA (Fig. 3b). Besides, the occurrences of AC, CA, AG and GA are observed to be positively associated with each other and negatively with the occurrences of other di-nucleotides (with some exceptions). Furthermore, the appearances of di-nucleotides CC, TC, GG, CG and GC are noticed to be positively correlated among themselves but negatively correlated with that of AA, TG, TT, AT and TA (Fig. 3b). As far as the distribution of tri-nucleotides are concerned, correlations are mostly positive within 32 tri-nucleotides in one group and 32 tri-nucleotides in the other group, whereas the correlations are mostly negative between these two groups (Fig. 3c).

**Figure 4:**
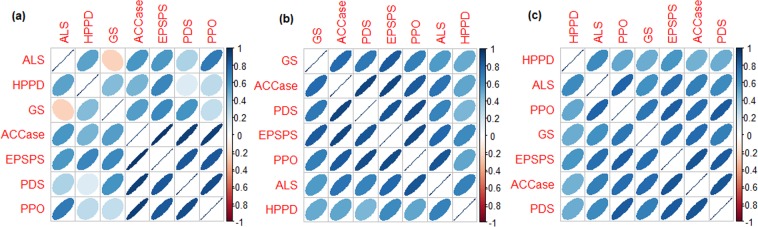
Heat maps of the correlations among the seven resistant classes, computed over (**a**) nucleotides, (**b**) di-nucleotides and (**c**) tri-nucleotides compositions.

All the seven resistant classes are found to be positively correlated with each other as far as the distribution of nucleotides (Fig. 4a), di-nucleotides (Fig. 4b) and tri-nucleotides (Fig. 4c) are concerned. However, the degrees of correlations are higher among ACCase, EPSPS, PDS and PPO, and lower among ALS, HPPD and GS as well as between these two groups (Fig. 4a–c).

### Performance analysis of binary classification

The CkM feature set is seen to be better as compared to the PkM feature set for classification of resistant and non-resistant classes, while comparison is made using a sample dataset with the combination of 4 K-mers i.e., K = 1, +2 + 3 + 4 (sub-section *feature and kernel analysis*). Thus, CkM feature set is preferred for subsequent analysis. Besides, the final prediction is also made with the remaining 14 possible combinations of the considered 4 K-mers (i.e., K = 1, K = 2, K = 3, K = 4, k = 1 + 2, K = 1 + 3, K = 1 + 4, K = 2 + 3, K = 2 + 4, K = 3 + 4, K = 1 + 2 + 3, K = 1 + 2 + 4, K = 1 + 3 + 4, K = 2 + 3 + 4). For all the 15 combinations of K-mers, estimates of performance metrics computed over 100 bootstrap sample sets (each set contains 120 resistant and 120 non-resistant sequences as mentioned in sub-section *Binary classification using balanced datasets*) along with 5 folds in each set are presented in Table 1. Though the sensitivity is not improved with the inclusion of tetramer features (K = 4), higher degree of improvement in specificity as well as in overall accuracy (Acc) is observed. In particular, Acc, Pre, MCC and AUC-ROC are observed to be increased by ~4%, ~8%, ~8% and ~7% respectively, after including 256 tetramer features in the other combinations of K-mer features. It is also found that with increase in the number of features, accuracies are not always increased. For instance, accuracies for K = 1 + 3 + 4 (324 features) are little higher than that of K = 2 + 3 + 4 (336 features). Furthermore, accuracies for K = 1 + 2 + 3 + 4 are seen to be higher than that of other combination of K-mers. Specifically, for K = 1 + 2 + 3 + 4 specificity (92%) is observed to be higher than the sensitivity (85%). Overall accuracy and AUC-ROC are found to be ~89%. Among all the performance metrics, precision is highest (92%) as well as most stable, whereas MCC is lowest (78%) and least stable (Table 1).

**Table 1:** Estimates of the performance metrics for classification of resistant and non-resistant categories under different combinations of K-mer features.

Sen	Spe	Acc	Pre	MCC	AUC-ROC	*K*-mer combination	#Features
0.9377 (±0.035)	0.015 (±0.010)	0.4763 (±0.017)	0.4875 (±0.009)	−0.1133 (±0.075)	0.4071 (±0.050)	K = 1	4
0.6201 (±0.097)	0.7539 (±0.074)	0.687 (±0.051)	0.7184 (±0.052)	0.3805 (±0.099)	0.528 (±0.116)	K = 2	16
0.7245 (±0.030)	0.7229 (±0.029)	0.7237 (±0.022)	0.7231 (±0.024)	0.4494 (±0.045)	0.5634 (±0.035)	K = 1 + 2	20
0.8706 (±0.014)	0.8189 (±0.020)	0.8448 (±0.015)	0.8282 (±0.020)	0.6909 (±0.029)	0.8039 (±0.020)	K = 3	64
0.8671 (±0.080)	0.8206 (±0.042)	0.8438 (±0.041)	0.8289 (±0.034)	0.6888 (±0.082)	0.7992 (±0.070)	K = 1 + 3	68
0.8676 (±0.028)	0.824 (±0.028)	0.8458 (±0.022)	0.8317 (±0.024)	0.6927 (±0.045)	0.8063 (±0.038)	K = 2 + 3	80
0.864 (±0.016)	0.8323 (±0.019)	0.8481 (±0.015)	0.8376 (±0.019)	0.6969 (±0.030)	0.8123 (±0.019)	K = 1 + 2 + 3	84
0.8583 (±0.029)	0.9176 (±0.027)	0.888 (±0.022)	0.9127 (±0.023)	0.7774 (±0.044)	0.8841 (±0.037)	K = 4	256
0.8565 (±0.015)	0.9161 (±0.019)	0.8863 (±0.013)	0.911 (±0.019)	0.7741 (±0.027)	0.8832 (±0.018)	K = 1 + 4	260
0.8568 (±0.014)	0.9196 (±0.018)	0.8882 (±0.013)	0.9145 (±0.018)	0.7781 (±0.026)	0.8849 (±0.015)	K = 2 + 4	272
0.8565 (±0.029)	0.9173 (±0.026)	0.8869 (±0.023)	0.9121 (±0.023)	0.7753 (±0.046)	0.8833 (±0.035)	K = 1 + 2 + 4	276
0.8557 (±0.016)	0.9208 (±0.018)	0.8883 (±0.014)	0.9156 (±0.018)	0.7783 (±0.028)	0.8862 (±0.020)	K = 3 + 4	320
0.8567 (±0.017)	0.9198 (±0.020)	0.8883 (±0.016)	0.9147 (±0.020)	0.7782 (±0.031)	0.8868 (±0.020)	K = 1 + 3 + 4	324
0.8514 (±0.014)	0.9177 (±0.019)	0.8845 (±0.014)	0.9121 (±0.019)	0.7709 (±0.029)	0.8841 (±0.018)	K = 2 + 3 + 4	336
0.8508 (±0.021)	0.9299 (±0.017)	0.8903 (±0.016)	0.9240 (±0.017)	0.7833 (±0.032)	0.8930 (±0.030)	K = 1 + 2 + 3 + 4	340

Values inside the brackets indicate standard errors.

### Comparative analysis with BLAST algorithms for binary classification

For comparison, performances of the BLAST algorithms and the proposed approach were evaluated using a balanced dataset having randomly drawn 120 resistant and 120 non-resistant sequences from the respective classes. Performance metrics of the BLAST algorithms with different word sizes computed over 5-folds CV (for e-values 0.1 and 1) are given in Table 2. With increase in the word size (K-mer size), though the false positives are seen to be declined (higher specificity) true positives are also observed to decreased (lower sensitivity) (Table 2). Further, true positives are higher (higher sensitivity) for the e-value 1 than 0.1 but the false positives are also higher for the e-value 1 (lower precision) as compared to e-value 0.1. In other words, with stringent e-value though the false positive rates are seen to be declined, the true positive rates are also observed to be declined (Table 2). Although the sensitivities of BLAST algorithms at e-value 1 are seen at par with the proposed approach, all other performance metrics are found higher for the proposed approach. In particular, overall accuracy (Acc) of the developed approach (90%) is observed ~20% higher as compared to the BLAST algorithms (Table 2). As far as performances of BLAST algorithms are concerned, accuracies are little higher for *dc-megablast* than the other two approaches (with few exceptions), for both the e-values. Thus, the BLAST algorithm may be equally efficient as the developed approach in determining the resistant genes but the number of false positives will be higher.

**Table 2:** Classification accuracies for the binary classification of herbicide resistant and non-resistant categories using different versions of BLAST algorithms with different e-values and word sizes (K-mer).

Algorithm	e-value	Word_Size (K-mer)	Sen	Spe	Acc	Pre	MCC
*blastn*	0.1	8	0.642	0.825	0.733	0.786	0.475
		9	0.625	0.842	0.733	0.798	0.478
		10	0.608	0.858	0.733	0.811	0.482
		11(Default)	0.6	0.875	0.738	0.828	0.494
		12	0.592	0.908	0.750	0.866	0.527
		13	0.583	0.908	0.746	0.864	0.520
		14	0.533	0.950	0.742	0.914	0.532
	1	8	0.950	0.242	0.596	0.556	0.272
		9	0.925	0.379	0.652	0.561	0.355
		10	0.875	0.375	0.625	0.583	0.289
		11(Default)	0.875	0.5	0.688	0.636	0.405
		12	0.833	0.542	0.688	0.645	0.392
		13	0.833	0.592	0.713	0.671	0.438
		14	0.817	0.650	0.733	0.700	0.473
*megablast*	0.1	8	0.581	0.850	0.716	0.791	0.448
		9	0.583	0.850	0.717	0.795	0.450
		10	0.592	0.858	0.725	0.807	0.467
		11(Default)	0.55	0.9	0.725	0.846	0.48
		12	0.575	0.860	0.718	0.831	0.447
		13	0.550	0.900	0.725	0.846	0.480
		14	0.550	0.933	0.742	0.892	0.523
	1	8	0.933	0.367	0.650	0.596	0.364
		9	0.925	0.383	0.654	0.600	0.367
		10	0.925	0.442	0.683	0.624	0.419
		11(Default)	0.883	0.525	0.704	0.65	0.437
		12	0.900	0.508	0.704	0.647	0.444
		13	0.867	0.525	0.696	0.646	0.417
		14	0.858	0.583	0.721	0.673	0.459
*dc-megablast*	0.1	11(Default)	0.600	0.883	0.742	0.837	0.504
		12	0.575	0.900	0.738	0.852	0.502
	1	11(Default)	0.842	0.592	0.717	0.673	0.448
		12	0.767	0.725	0.746	0.736	0.492
Proposed	NA	NA	0.85	0.95	0.90	0.944	0.804

Standard errors are not reported because the performance metrics are computed by summing over all the 5-folds of cross validation instead of taking average over five folds.

### Comparative analysis with HMM for binary classification

**Figure 5:**
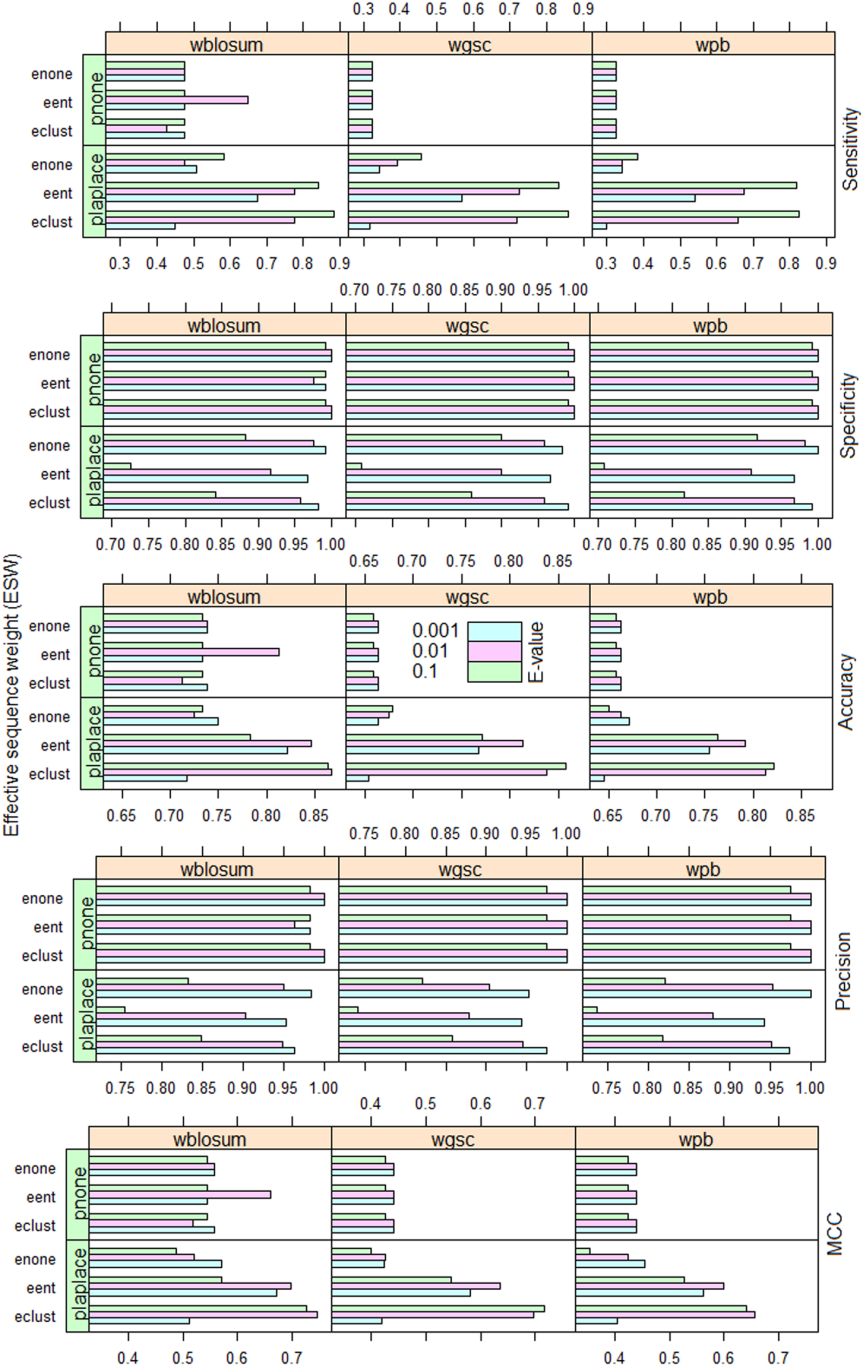
Bar plots of the performance metrics of HMM with respect to classification of herbicide resistant and non-resistant genes, with different combination of parametric values i.e., e-value (0.1, 0.01 and 0.001), controlling priors (*pnone* and *plaplace*), effective sequence weight (*eent*, *eclust* and *enone*) and relative sequence weight (*wpb*, *wgsc* and *wblosum*).

Estimates of performance metrics computed over 5-folds of the CV are shown in Fig. 5. For the relative sequence weights (RSW) *wpg* and *wgsc*, performance metrics are found to be almost same (fractional difference is not visible) for the controlling prior *pnone* with all the combinations of e-values and effective sequence weights (ESW). Similar to the BLAST algorithms, true positive rates (sensitivity) are seen to be declined with the stringency in the e-value but the true negative rates (specificity) are increased, for all the combination of RSW and ESW. For some combinations of parameters, true positive rates of the HMM are found at par with the proposed approach, whereas true negative rates are observed to be very low as compared to the proposed one for the same parametric combinations. Hence, the proposed approach and HMM may be equally efficient in detecting the true negatives but the efficiency of the proposed approach will be more for identifying the true positives. Thus, information on true positives may be lost if HMM is used for the prediction of GETS. The highest overall accuracy of the HMM (~86%) is found for the parameter combination ESW(*eclust*)-RSW(*wblosum*)-prior(*plaplace*)-evalue(0.01), which is ~3% less than the proposed approach (89%, kindly refer Table 1 for K = 1 + 2 + 3 + 4). For this parametric combination, MCC (~75%) of HMM is also ~3% less than that of proposed model (78.3%). However, precision of the HMM (~95%) is seen ~3% higher than the proposed one (92.4) and this may be due to the higher values of specificity for HMM. Considering all the performance metrics, proposed model achieved a little higher accuracy than the HMM. Nevertheless, the developed computational model will supplement the HMM algorithm for identification of GETS.

### Comparative analysis of supervised learning techniques for binary classification

**Figure 6:**
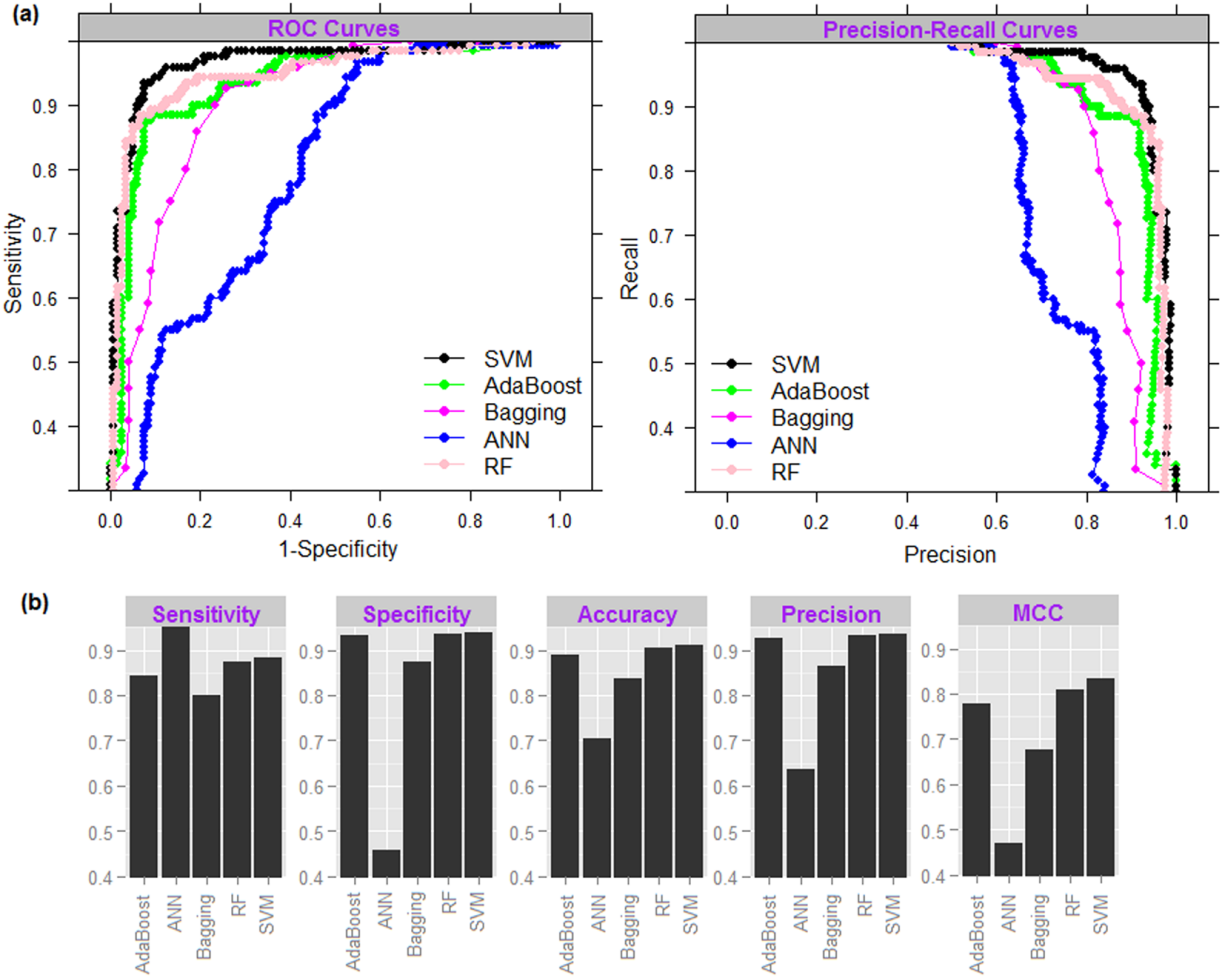
(**a**) ROC and PR curves, and (**b**) bar plots of the performance metrics for different machine learning classifiers for the classification of herbicide resistant and non-resistant genes. It can be seen that the SVM performed better as compared to the other classifiers in terms of all the performance measures.

Performances of the supervised learning techniques are measured with the same balanced dataset (120 positive and 120 negative sequences) that was used for evaluating the BLAST algorithms and HMM. From PR and ROC curves (Fig. 6a), higher accuracy is observed for SVM than that of other four learning algorithms. Further, overall accuracy (Acc) is lowest for ANN (0.704%) and is highest for SVM (91.1%) followed by RF (90.6%) (Fig. 6b). The sensitivity (88.2%), specificity (94%) and overall accuracy (91.1%) of SVM are little higher than that of RF (87.5%, 93.7%, 90.6%) but they are not found to be significantly different (Pval >0.05, Mann-Whitney test) from each other (Fig. 6b). As far as MCC is concerned, >80% MCC is achieved by SVM and RF, whereas it is up to 80% for rest of the classifiers. Among the ensemble learning methods, accuracy is higher for RF as compared to AdaBoost, and AdaBoost performed better than the Bagging classifier. We preferred SVM for subsequent analysis because of its higher accuracy.

### Analysis of one-to-one discrimination

In this discrimination, each resistant category was discriminated from rest of the six resistant categories. With the combination of CkM features and SVM classifier, PPO and PDS categories are discriminated from the remaining six categories with 100% accuracy (Table 3). It is also observed that the overall accuracy for each category is >97% (Table 3). In particular, specificity and sensitivity for each resistant category is >91% and >97% respectively. Except HPPD, precision (Pre) and MCC are also found to be >97% and >93% respectively (Table 3). This implies that not only the resistant class can be discriminated from non-resistant class but also each resistant class can be discriminated from other resistant classes with higher accuracy.

**Table 3:** Prediction accuracies of the developed model for discriminating each resistant category form rest of the 6 resistant classes, while accuracies are measured through jackknife validation.

Category	Sen	Spe	Acc	Pre	MCC
ACCase	1.000	0.993	0.995	0.973	0.983
ALS	0.919	0.993	0.978	0.971	0.931
EPSPS	0.931	1.000	0.989	1.000	0.959
GS	0.920	1.000	0.989	1.000	0.953
HPPD	1.000	0.970	0.973	0.783	0.871
PDS	1.000	1.000	1.000	1.000	1.000
PPO	1.000	1.000	1.000	1.000	1.000

Standard errors are not reported as metrics are computed over jackknife cross validation.

### Two-stage prediction analysis for independent dataset

**Figure 7:**
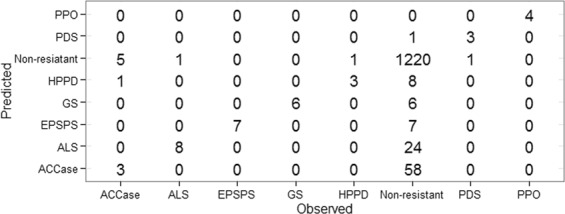
Confusion matrix for the number of correctly and wrongly predicted observations of the independent dataset, using two-stage prediction model.

For prediction of the independent dataset, the binary classifier trained with 120 resistant and 120 non-resistant sequences (same as the dataset used to evaluate blast algorithms, HMM and supervised learning techniques) was in the first stage, whereas the multiclass classifier trained with 36 ACCase, 37 ALS, 29 EPSPS, 25 GS, 18 HPPD, 18 PDS and 20 PPO (as mentioned in *One-to-one discrimination*) was used in the second stage. Here, the independent dataset contains 1324 non–resistant (excluding 120 non-resistant sequences used to train the binary classifier in the first stage) and 44 resistant sequences (as mentioned in sub-section *Acquisition of herbicide resistant and non-resistant sequence dataset*). Using the two stage prediction process, all the instances of PPO, EPSPS and GS are correctly predicted into their respective categories. On the other hand, one sequence from each of ALS, HPPD and PDS is misclassified into the non-resistant category (Fig. 7). It is also seen that the number of misclassified observations are higher for ACCase than the other categories. Specifically, 36 (out of 44) resistant and 1221 (out of 1324) non-resistant observations are correctly classified (Fig. 7), and hence the overall accuracy for the independent dataset is ~87% ($\frac{1}{2}(\frac{36}{44}+\frac{1220}{1324})=0.8698$). Interestingly, it is noticed that none of the sequences of any resistant category are misclassified into another resistant category. Furthermore, non-resistant sequences are mostly misclassified into ACCase category, whereas no misclassification is observed for non-resistant sequences into PPO category.

### Single-stage prediction analysis for independent dataset

To compare with the two-stage prediction, independent dataset was also predicted based on a single multiclass-classifier (i.e., single stage prediction). Here, training of the prediction model was done with 8 different classes i.e., seven resistant and one non-resistant. In particular, the multi-class classification model was trained with 18 ACCase, 22 ALS, 22 EPSPS, 19 GS, 15 HPPD, 13 PDS, 11 PPO and 120 non-resistant sequences. Out of 44 resistant and 1324 non-resistant sequences of the independent dataset, 32 and 1287 were correctly predicted into their respective classes. Hence, the overall accuracy with the single stage prediction model is $\frac{1}{2}(\frac{32}{44}+\frac{1287}{1324})=0.8496$, which is ~2% less than that of two-stage prediction (0.8698). Thus, there is a probability of under prediction for the resistant class if single stage prediction is preferred for the independent dataset. Therefore, a two stage prediction process may be useful over the single stage multiclass-classifier (if non-resistant category has to be used as one of the classes).

### Online prediction server: HRGPred

To facilitate easy computational identification of GETS using the proposed model, we have established an online prediction server HRGPred. The prediction in HRGPred is made in two stages: (i) the sequences are predicted as resistant or non-resistant in the first stage and (ii) the sequences predicted as resistant in the first stage are subjected to the second stage, where they are classified into any one of the seven resistant classes. The HRGPred has been trained with 120 resistant and 120 non-resistant cDNA sequences in the first stage and seven resistant classes (36 ACCase, 37 ALS, 29 EPSPS, 25 GS, 18 HPPD, 18 PDS and 20 PPO) in the second stage. The results are presented in tabular form that includes sequence number, identifiers, predicted class and probabilities. The HRGPred can be freely accessed via http://cabgrid.res.in:8080/hrgpred. All the sequence datasets are maintained at http://cabgrid.res.in:8080/hrgpred/dataset.html for reproducibility of the work.

## Discussion

Herbicide resistance is not only a threat to the agriculture^82^, but also has negative impact on the biodiversity^83,84^. By January 2017, 478 biotypes have been reported to develop resistance, encompassing 23 sites of action and 161 herbicides^85,86^. Although the genetic factors significantly contribute to the evolution of HR^30^, it is difficult to predict exactly which weed species will have the biotypes resistant to a given herbicide. However, getting into the genetics insight of HR may be helpful to delay the process of evolution and distribution of HR. To supplement this understanding, this study presents a computational model for the identification of seven categories of GETS i.e., ACCase, EPSPS, ALS, GS, HPPD, PPO and PDS.

Prediction accuracy may be inflated, if unbalanced dataset is used for training of the machine learning-based computational model^87,88^. Thus, the balanced dataset was used to evaluate the proposed prediction model. Besides, the sequence similarities in resistant and non-resistant datasets were also kept at <90% and <60% respectively to avoid over prediction^38^.

Two types of classifications were performed. First, classification between resistant and non-resistant class was made, where an overall accuracy of ~89% was found. Second, a multi-class classifier was built to discriminate each resistant category from rest of the six resistant categories, and an overall accuracy of >97% was found for each resistant category. Thus, it may be inferred that not only the GETS can be distinguished from the genes that do not encode herbicide target sites, based on the compositions of mono-, di- and tri-nucleotides but also the seven classes of GETS can be discriminated from each other with higher accuracy. Further, a pattern between the correlation and accuracy was noticed for different classes of GETS. Specifically, accuracies were lower for the classes with lower degrees of correlations (ALS, GS and HPPD) and higher for rest of the four highly correlated categories (Fig. 3).

Since BLAST is widely used analytical tool for searching the sequence homologs^89,90^, it was employed for the prediction of resistant and non-resistant genes. True positive rates of BLAST algorithms (at e-value 1) were found at par with the developed computational model but false positive rates were much higher in BLAST algorithms for both the e-values (0.1 and 1). Thus, use of BLAST algorithm for the prediction of herbicide resistant genes may result in the loss of information on true positives.

Profile-based methods often perform better than the pair-wise-based approaches like BLAST algorithms^91^. Further, HMM yields best result among the profile-based methods^92,93^. Thus, the HMM was also employed for prediction of HR genes with different combinations of parametric values. It was found to be equally efficient with the proposed model in detecting the non-resistant class but the developed computational model achieved much higher accuracy for predicting the resistant class. In other words, information on true positives may be lost if HMM is used for prediction. Thus, the HMM may not be suitable for prediction of GETS in spite of its successful application in other areas of computational biology^94,95^. Furthermore, HMM was found to achieve higher accuracy than the BLAST algorithms.

The HMM and BLAST algorithms were employed for the binary classification only (classification of resistant and non-resistant class) and not for the multi-class classification (which is second stage of classification). Because, jackknife cross validation (leave-one-out cross validation) analysis was used for the second stage classification (discrimination of seven resistant classes from each other) and it does not seem to be feasible in case of BLAST and HMM. Another reason for not performing the second stage classification using BLAST and HMM in this study is the low classification accuracy of comparison algorithms (BLAST and HMM) in first stage as compared to the SVM-based model.

Besides homology based algorithms (BLAST and HMM), classification was also made with other state-of-art supervised learning techniques viz., Bagging, AdaBoost, ANN and RF. Among these classifiers, lowest accuracy was observed for ANN. This may be due to the fact that except ANN, other three classifiers (Bagging, AdaBoost and RF) are ensemble learning methods that have been reported to perform better than a single classifier^96–100^. Further, performances of the ensemble classifiers were found in the order of RF >AdaBoost >Bagging, which is an expected trend because AdaBoost is an improvement over Bagging and RF is an improvement over AdaBoost classifier^76^.

Inspired by the earlier studies^38,88,101^, prediction for the independent dataset was carried out in two stages. Higher misclassification rates were observed for ACCase, whereas all EPSPS, GS and PPO were correctly classified. In particular, sequences of the ACCase were found to be misclassified in non-resistant class and vice-versa, which may be due to a higher degree of sequence similarity between ACCase and non-resistant class. Further, few instances of the non-resistant class were found to be misclassified in EPSPS, GS, PDS, HPPD and PPO. This implies that there may be a lesser degree of similarity between the resistant (EPSPS, GS, PDS, HPPD and PPO) and non-resistant class. To compare the performance of two-stage prediction model with single-stage prediction model, prediction for the independent dataset was further made using a single multiclass classifier trained with 8 classes (one non-resistant and seven resistant). The overall accuracy was found to be little higher in two-stage prediction model as compared to the single-stage model. We believe that in the proposed model the prediction accuracy was improved by the contribution of both feature generation and SVM algorithm. Because, (i) accuracies were observed to be higher for the K-mer combination K = 1 + 2 + 3 + 4 as compared to the other 14 combinations of K-mer features (Table 1), and (ii) SVM algorithm was found to achieve higher accuracy as compared to the other supervised learning techniques (Fig. 6).

Development of a user-friendly and freely available prediction tool for any theoretical approach is not only useful for a large section of experimental scientists but also represents future direction for the development of such computational tools^81,102^. Thus, we have established a web server HRGPred for the prediction of seven classes of GETS based on the developed computational approach. We expect that the HRGPred will certainly aid to the prevailing efforts for annotation of the genes related to herbicide resistance.

## Data Availability

All the datasets used in this study are freely accessible at http://webapp.cabgrid.res.in/hrgpred/dataset.html.
